# Supplementary material for: Social and structural determinants associated with the prevalence of sexually transmitted infections among female commercial sex workers in Dhaka City, Bangladesh
Source: PLOS Glob Public Health. 2024 Jan 18;4(1):e0002797. doi: 10.1371/journal.pgph.0002797 (PMC10796017; doi:10.1371/journal.pgph.0002797)
Supplement: S1 Text — (DOCX) [file pgph.0002797.s001.docx]

**Supplementary File S1 Text**

**S1 Text:** Texts containing additional details on the different ‘categories’ of independent variables and Estimation of consistent condom use (CCU) (**Materials and Methods section**)

**2 Materials and Methods**

**2.3 Measures**

***Independent-variables:*** To determine the structural relationship with STI-prevalence, authors followed ‘HIV risk-environmental framework’ proposed by Shannon and colleagues [39,40]. Thus, collected data were broadly segmented into three major domains under which macro/micro structural factors remain at physical/social/economic/policy levels: **(1) socio-economic and Individual risk factors (Table 2)**: Factors/reasons for being sex workers (FCSWs) (Poverty/deception/others), Internal migration (Migrated from other cities, yes/no), Seeking customers in another city ( yes/no), Weekly income (<7000 or ≥7000-BDT), Income shared with others (Yes/no), Present age (≤18 y/18.1-29.9 y/≥30 years), Introductory age (≤18 y/18.1-29.9 y/ ≥30 years), Education [(No schooling/1-12 years schooling (1-5 years for primary, 6-8 years for junior, 9-10 years for secondary, and 10-12 years for higher secondary)], Marital status (Married/unmarried or widowed/divorced/abandoned), Years in the sex trade (<1 y/1-5 y/≥6 years), Family members were informed about professional sex work (Yes/no), Smoking (Yes/no), Alcohol consumption (Yes/no), Abuse substances (Yes/no), Use contraceptives (Never/pills/condoms/other methods), STI-symptoms (Having all/1-2 symptoms/No), STI knowledge (Yes/no), Self-reported HIV-risk perception (No/low/high)] **(2)** **Sexual networking and High-risk sex behaviors/HRSBs (Table 3)**: Having a current non-paying sexual partners (NPSPs) (Yes/no), Monthly-coitus with Regular clients (1-2 times/≥3 times/having no regular clients), Clients per day (1-14 clients/≥15), Group sex (GS) ever (Yes/no), Heterosexual anal intercourse (HAI)/Anal sex ever (Yes/no), last week’s oral sex/OS: fellatio/cunnilingus (Yes/no), Condom use (Never/Inconsistent or consistent)] **(3)** **work environment (Table 3)**: Places of the sex trade (Roads/parks/shrines/hotels/markets), Problem faced for being sex workers (Lifetime problems) (Yes/no), last year’s police arrestment (Yes/no), last year’s forced-sex (Yes/no), Membership in FCSW’s organization (Yes/no), Controlled by Pimps/Dalal (Yes/no), Access to condoms/condom collected from (Colleagues/hotel boys/Self-buying/NGOs), and HIV-testing ever (Yes/no). **Important to note that** factors such as Introductory age, Alcohol consumption, Substance abuses, STI-symptoms and Knowledge, Clients per day, Counseling on STIs/HIV and Access to STI-treatment were non-significant (P>0.05) at bivariate analysis, and were not included in the tables (Table 2, and Table 3) of the manuscript.

**Estimation of consistent condom use (CCU)**

‘Consistent condom use’ (CCU) was estimated from positive answers of the seven questions in the questionnaire where FCSWs were asked ‘whether condom used consistently with the last sexual encounter for all commercial/non-commercial clients/partners’. These seven questions were as follows: **(1)** Consistent condom use in Anal sex or heterosexual anal intercourse/HAI-CCU **(2)** Consistent condom use in group sex/GS-CCU, **(3)** Consistent condom use in oral sex/OS (Fellatio-CCU), **(4)** Consistent condom use with normal/casual/one-time clients **(5)** Consistent condom use with regular clients (6) Consistent condom use with non-paying sex partners/NPSPs-CCU (7) and lastly by the question ‘what measure was followed to stay away from STIs/HIV?’, If one answered 'Yes' for all questions 1-7, that denoted she used condom ':Consistently'; if someone failed once, that was 'Inconsistent,' and if FCSWs answered ‘No’ for all seven questions, was denoted as 'Never.'
