# Supplementary material for: Social and structural determinants associated with the prevalence of sexually transmitted infections among female commercial sex workers in Dhaka City, Bangladesh
Source: PLOS Glob Public Health. 2024 Jan 18;4(1):e0002797. doi: 10.1371/journal.pgph.0002797 (PMC10796017; doi:10.1371/journal.pgph.0002797)
Supplement: S1 Table — (DOCX) [file pgph.0002797.s002.docx]

**S1 Table:** Bivariate analysis (unadjusted odds ratio/UOR) shows different structural determinants associated with the prevalence of STIs among female sex workers.

| **Structural determinants (Micro/Macro)** | | | **All Prevalence of STIs** | | | **Unadjusted Odds ratio (UOR)** |
| --- | --- | --- | --- | --- | --- | --- |
| **Structural Environment** | **Socio-economic variables** | | **%**  **(N=495)** | **No**  **(n=279)** | **Yes (n=216)** | **95% CI (lower-upper)** |
| Macro-physical | Reasons for being sex workers | |  |  |  |  |
|  | Poverty^R^ | | 55.8 (276) | 60.9 (170) | 49.1 (106) |  |
|  | Exploitation | | 26.1 (129) | 23.7 (66) | 29.2 (63) | 1.5 (1.0-2.3)^*^ |
|  | ^a^Others | | 18.1 (90) | 15.4 (43) | 21.8 (47) | 1.8 (1.1-2.8)^*^ |
| Macro-social | Migrated in Dhaka from other city | | 85.3 (422) | 82.4 (230) | 88.9 (192) | 1.7 (1.0-2.9)^*^ |
|  | No^R^ | | 14.7 (73) | 17.6 (49) | 11.1 (24) |  |
|  | Didn’t Seek customers in another city^R^ | | 85.3 (422) | 82.4 (230) | 88.9 (192) |  |
|  | Yes | | 14.7 (73) | 17.6 (49) | 11.1 (24) | 1.15 (.707-1.87)^*^ |
| Micro-economic | ^b^Income (weekly) | |  |  |  |  |
|  | <7000 (BDT) | | 72.1 (357) | 64.2 (179) | 82.4 (178) | 3.5 (2.1-5.7) ^**^ |
|  | ≥7000^R^ | | 22.8 (113) | 31.5 (88) | 11.6 (25) |  |
|  | *^c^*Income sharing (Forcefully/willingly) | | 28.1 (139) | 26.5 (74) | 30.1 (65) | 1.1 (.804-1.77) |
|  | No^R^ | | 71.9 (356) | 73.5 (205) | 69.9 (151) |  |
| Micro-physical | **Individual/behavioral risks factors** | |  |  |  |  |
|  | ^d^Present age (Years) | |  |  |  |  |
|  | ≤18 | | 17.8 (88) | 26.9 (75) | 6.0 (13) | .08 (.05-.19)^*^ |
|  | 18.1-29.9 | | 46.3 (229) | 50.2 (140) | 41.2 (89) | .36 (.24-.54) |
|  | ≥30^R^ | | 36.0 (178) | 22.9 (64) | 52.8 (114) |  |
|  | Introductory age (years) | |  |  |  |  |
|  | 10-17 | | 43.6 (215) | 41.9 (117) | 45.4 (98) | .89 (.557-1.14) |
|  | 18-24 | | 34.9 (173) | 38.4 (107) | 30.6 (66) | .652 (.401-1.1) |
|  | ≥25^R^ | | 21.5 (107) | 19.7 (55) | 24.1 (52) |  |
|  | Education (year) | |  |  |  |  |
|  | No schooling | | 69.5 (344) | 59.5 (166) | 82.4 (178) | 3.2 (2.1-4.9) ^**^ |
|  | ^e^Schooling years 1-12^R^ | | 30.5 (151) | 40.5 (113) | 17.6 (38) |  |
|  | Marital status | |  |  |  |  |
|  | Married | | 41.6 (206) | 39.8 (111) | 44.0 (95) | .88 (.60-1.2) |
|  | Unmarried | | 13.7 (68) | 20.1 (56) | 5.5 (12) | .22 (.11-.43) ^**^ |
|  | ^f^Widowed/divorced/abandoned^R^ | | 44.6 (221) | 40.1 (112) | 50.5 (109) |  |
|  | Years in the sex-trade | |  |  |  |  |
|  | <1 | | 10.7 (53) | 16.5 (46) | 3.2 (07) | .07 (.03-.17)^**^ |
|  | 1-5 | | 52.1 (258) | 62.4 (174) | 38.9 (84) | .23 (.152-.341)^*^ |
|  | ≥6^R^ | | 37.2 (184) | 21.1 (59) | 57.9 (125) |  |
|  | Family members knowing about professional sex work | 41.4 (205) | | 34.1 (95) | 50.9 (110) | 2.0 (1.4-2.89)^**^ |
|  | No^R^ | | 58.6 (290) | 65.9 (184) | 49.1 (106) |  |
|  | Smoking^R^ | | 42.6 (211) | 38.7 (108) | 47.7 (103) |  |
|  | No | | 57.4 (284) | 61.3 (171) | 52.3 (113) | .69 (.484-.993)^*^ |
|  | Drink Alcohol | | 11.1 (55) | 12.5 (35) | 9.3 (20) | 1.4 (.787-2.51) |
|  | No^R^ | | 88.9 (440) | 87.5 (244) | 90.7 (196) |  |
|  | Abuse substances | |  |  |  |  |
|  | Yes Cannabis | | 11.1 (55) | 9.3 (26) | 13.4 (29) | 1.5 (.896-2.77) |
|  | ^g^Others | | 7.5 (37) | 6.1 (17) | 9.3 (20) | 1.6 (.845-3.27) |
|  | No^R^ | | 81.4 (403) | 84.6 (236) | 77.3 (167) |  |
|  | ≥1 abortion-cases^R^ | | 27.1 (134) | 21.1 (59) | 34.7 (75) |  |
|  | No | | 72.9 (361) | 78.9 (220) | 65.3 (141) | .50 (.34-.75) |
|  | Used Contraceptives | |  |  |  |  |
|  | Never | | 16.8 (83) | 15.4 (43) | 18.5 (40) | 1.5 (.54-2.24)^*^ |
|  | Pill | | 30.7 (152) | 37.6 (105) | 21.8 (47) | .53 (.27-1.0) |
|  | Condom | | 42.8 (212) | 37.6 (105) | 49.5 (107) | 1.3 (.64-2.26)^*^ |
|  | ^h^Others^R^ | | 9.7 (48) | 9.3 (26) | 10.2 (22) |  |
|  | Having STI-symptoms^R^ | | 48.1 (238) | 46.2 (129) | 50.5 (109) |  |
|  | No | | 51.9 (257) | 53.8 (150) | 49.5 (107) | .84 (.592-1.21) |
|  | Self-reported HIV-risk | |  |  |  |  |
|  | No | | 52.3 (259) | 49.5 (138) | 56.1 (121) | .74 (.47-1.2) |
|  | Low | | 27.9 (138) | 34.4 (96) | 19.4 (42) | .07 (.22-.40)^**^ |
|  | High^R^ | | 19.8 (98) | 16.1 (45) | 24.5 (53)  () |  |

^a^Anger (on family members/husband)/Drug-addiction/self-motivation/raped/tortured; ^b^Didn’t work 5.0% (n=25);  *^c^*Local muscle-man/Goons, family, police, others (Pimps/lovers/partners/brother-in-laws); ^d^Mean age: 26.5±7.9 (14-55); ^e^Primary (1-5 Y) 26.9% (n=129) & Secondary (6-12 Y) 4.4% (n=22); ^f^Abandoned means marital state when husband remains absconding/leaving wife illegally; ^g^Dry cough Linctus (Phensidyl cough syrup)/Dried tobacco dust on gum (smokeless) etc.; ^h^Ligation/menopause/modern methods (Nor-plant/copper-T/Injection);  ^R^Reference category, ^*^P<0.05 ^**^P<0.001

**Supplementary S1 Table-continued**

| **Structural Environment** | **Sexual networking, high-risk sexual behaviors (HRSBs) UOR** | | | | | | |
| --- | --- | --- | --- | --- | --- | --- | --- |
| Micro-Social | Having a nonpaying current partner^R^ | | | | 10.9 (54) | 13.6 (38) | 7.4 (16) |
|  | No | | | | 89.1 (441) | 86.4 (241) | 92.6 (200) |
|  | Monthly coitus with regular clients | | | |  |  |  |
|  | 1-2-times | | | 20.2 (100) | 20.8 (58) | 19.4 (42) | .76 (.484-1.19) |
|  | ≥3-times | | | 13.9 (69) | 19.4 (54) | 6.9 (15) | .29 (.158-.538)^**^ |
|  | No clients^R^ | | | 65.9 (326) | 59.9 (167) | 73.6 (159) |  |
|  | Performed group sex-ever | | | 25.9 (128) | 20.8 (58) | 32.4 (70) | 1.8 (1.21−2.7)^*^ |
|  | No^R^ | | | 74.1 (367) | 79.2 (221) | 67.6 (146) |  |
|  | Anal sex-ever^R^ |  | 6.7 (33) | | 3.6 (10) | 10.6 (23) |  |
|  | No | | | 93.3 (462) | 96.4 (269) | 89.4 (193) | .31 (.15-.67)^**^ |
|  | Last-week oral sex Fellatio) | | | 12.3 (61) | 13.3 (37) | 11.1 (24) | 1.2 (.71-2.12) |
|  | No^R^ | | | 87.7 (434) | 86.7 (242) | 88.9 (192) |  |
|  | Last-week-Oral sex (Cunnilingus) | | | 17.0 (84) | 19.0 (53) | 14.4 (31) | 1.4 (.86-2.27) |
|  | No^R^ | | | 83.0 (411) | 86.7 (226) | 85.6 (185) |  |
|  | Condom use | | |  |  |  |  |
|  | ^h^Never/inconsistent ^R^ | | | 54.1 (268) | 41.9 (117) | 69.9 (151) |  |
|  | ^i^Consistent condom use | | | 45.9 (227) | 58.1 (162) | 30.1 (65) | .31 (.214-.453)^**^ |

|  | **Work environment** | | | | | |  |  | |
| --- | --- | --- | --- | --- | --- | --- | --- | --- | --- |
| Micro-physical | Places of the sex-trade | | |  | |  |  | |  |
|  | Road/Park/shrine/Market | | | 64.0 (317) | | 50.5 (141) | 81.5 (176) | | 4.3(2.84−6.52)^**^ |
|  | Hotel^R^ | | | 36.0 (178) | | 49.5 (138) | 18.5 (40) | |  |
| Macro-social-policy | ^j^Faced-Problems for being a sex workers (lifetime) | | | 81.2 (402) | | 74.9 (209) | 89.4 (193) | | 4.9 (2.1-12.1)^**^ |
|  | No^R^ | | | 18.8 (93) | | 25.2 (70) | 10.6 (23) | |  |
|  | Police-arrestment (last 1-year) | | | 34.7 (172) | | 26.9 (75) | 44.9 (97) | | 2.2 (1.52-3.23)^**^ |
|  | No^R^ | | | 65.3 (323) | | 73.1 (204) | 55.1 (119) | |  |
|  | ^k^Forced-sex (last 1-year) | | | | 47.3 (234) | 39.4 (110) | 57.4 (124) | | 2.1 (1.44-2.97) ^**^ |
|  | No^R^ | | | 52.7 (261) | | 60.6 (169) | 42.6 (92) | |  |
|  | Membership (FCSW’s organization) | | | 39.6 (196) | | 39.8 (111) | 39.4 (85) | | .98 (.68-1.41) |
|  | No^R^ | | | 60.4 (299) | | 60.2 (168) | 60.6 (131) | |  |
| Micro-policy | Controlled by Pimps/Dalal | | | 7.1 (35) | | 6.5 (18) | 7.9 (17) | | 1.2 (.623-2.47) |
|  | No^R^ | | | 92.9 (460) | | 93.5 (261) | 92.1 (199) | |  |
|  | Knowledge on STI-symptoms | | |  | |  |  | |  |
|  | ^l^known all | | | 41.8 (207) | | 39.1 (109) | 45.4 (98) | | .93 (.556-1.56) |
|  | 1/or 2 | | | 16.8 (83) | | 17.2 (48) | 16.2 (35) | | 1.2 (.737-2.1) |
|  | Not-any^R^ | | | 41.4 (205) | | 43.7 (122) | 38.4 (83) | |  |
|  | STI/HIV-Counseling by NGOs (last-6 months) | | | **48.9 (242)** | | **50.5 (141)** | **46.8 (101)** | |  |
|  | *Advised to go to Clinics* | | | *50.0 (121)* | | *47.5 (67)* | *53.5 (54)* | | *2.1 (.787-5.86)* |
|  | *Given condom (free)* | | | *21.1 (51)* | | *19.2 (27)* | *23.8 (24)* | | *2.3 (.799-7.03)* |
|  | |  | *Trained how to use condom* | *19.8 (48)* | | *22.0 (31)* | *16.8 (17)* | | *1.4 (.482)* |
|  | |  | *Trained on HIV/AIDS*^R^ | *9.1 (22)* | | *11.3 (16)* | *5.9 (06)* | |  |
|  | | Access to STI-treatment | |  | |  |  | |  |
|  | |  | Didn’t know^R^ | 24.4 (121) | | 24.4 (68) | 24.5 (53) | |  |
|  | |  | (Yes) | **75.6 (374)** | | **75.6 (211)** | **75.5 (163)** | |  |
|  | |  | *Private clinic* | *16.4 (81)* | | *18.3 (51)* | *13.9 (30)* | | *.75 (.424-1.34)* |
|  | |  | *NGO clinic* | *52.3 (259)* | | *51.2 (243)* | *53.7 (116)* | | *1.0 (.674-1.61)* |
|  | |  | *^m^Others* | *6.9 (34)* | | *6.1 (17)* | *7.9 (17)* | | *1.3 (.599-2.75)* |
|  | | Access to condom/condom collected from (n=439) | |  | |  |  | |  |
|  | |  | Colleagues^R^ | 23.0 (101) | | 21.1 (54) | 25.7 (47) | |  |
|  | |  | Hotel boys | 29.2 (128) | | 41.4 (106) | 12.1 (22) | | .23 (.130-.436)^**^ |
|  | |  | Self-buying | 22.1 (97) | | 15.6 (40) | 31.1 (57) | | 1.6 (.933-2.87) |
|  | | NGOs | | 25.7 (113) | | 21.9 (56) | 31.1 (57) | | 1.1 (.683-2.0) |
|  | | HIV-testing (ever) | | 30.9 (153) | | 23.7 (66) | 40.3 (87) | | 2.2 (1.48-3.2)^**^ |
|  | |  | No^R^ | 69.1 (342) | | 76.3 (213) | 59.7 (129) | |  |

^h^Never used 11.3% (n=56), inconsistent/occasional 42.8% (n=212); ^i^When FCSWs used condom at her every sexual encounter [(e.g. vaginal intercourse (VI), heterosexual-anal intercourse (HAI), group sex (GS), fellatio (Oral-Sex)] with every clients/partner [e.g. permanent client (PC), occasional client (OC), non-paying sex-partners (NPSP)]); ^j^Police-harassment/people’s bad-remarks/others (clients’ didn’t pay/family’s disliking/forced-sex/forced not to use condoms/funeral-problem, always tensed); ^K^by local musclemen/police/customers/others (hotel-boys/manager/lovers/brother-in-laws); ^l^Smelly-vaginal-discharge, vaginal scar & lower-abdominal pain without menstruation; ^m^Public hospitals/drug dispensary/private doctor etc.; Non-government organizations=NGOs; ^R^Reference category ^*^P<0.05 ^**^P<0.001
