# Supplementary material for: Social and structural determinants associated with the prevalence of sexually transmitted infections among female commercial sex workers in Dhaka City, Bangladesh
Source: PLOS Glob Public Health. 2024 Jan 18;4(1):e0002797. doi: 10.1371/journal.pgph.0002797 (PMC10796017; doi:10.1371/journal.pgph.0002797)
Supplement: Attachment — Submitted filename: Response_To_Reviewers_(Rebuttal_Letter).docx [file pgph.0002797.s004.docx]

May 20, 2022

Editors-in-Chief

PLOS Global Public Health

Subject: Re-submission of the manuscript

Title:	“**Social and Structural Determinants Associated with the Prevalence of Sexually	Transmitted Infections among Female Commercial Sex Workers in Dhaka City,	Bangladesh”**

Dear [Kyobutungi](https://journals.plos.org/globalpublichealth/s/editors-in-chief#loc-catherine-kyobutungi) and [Pai](https://journals.plos.org/globalpublichealth/s/editors-in-chief#loc-madhukar-pai)

We are resubmitting the above mentioned ‘Main revised manuscript’ along with a ‘Point-to-point’ response letter (on the following pages) to the distinguished editors and reviewers and a highlighted ‘Manuscript with track changes’ (separate file) for publication in the ‘PLOS Global Public Health’ journal. According to the editorial decision and reviewer’s suggestions this revised manuscript is copy-edited by Ms. Meherun Nessa, Associate Professor (English), Department of Language, Sher-e-Bangla Agricultural University, Dhaka-1207 (E-mail: [meherun17@yahoo.com](mailto:meherun17@yahoo.com)).

We confirm that this work is original and has not been published previously, not currently in press, not publicly available in any form or format, nor is it currently under consideration for publication elsewhere and all authors have approved of and have agreed to submit the revised manuscript to this journal. Authors have declared no conflict of interest.

Sincerely

Mahbuba Kawser

Institute of Nutrition and Food Science,

University of Dhaka-1000, Bangladesh**.**

Cell: +880-1715095605

E-mail: [mahbubakawser@gmail.com](mailto:mahbubakawser@gmail.com)

**Table: Point to point responses to reviewer’s comments about the manuscript** “Social and Structural Determinants Associated with the Prevalence of Sexually Transmitted Infections among Female Commercial Sex Workers in Dhaka City, Bangladesh”

| Journal requirements | Explanation/description | Remarks |
| --- | --- | --- |
| 1. Co-authors’ non-responsiveness to the e-mails   (Co-authors have not confirmed authorship of the manuscript. We have resent them the authorship confirmation email; however please check that the above email address for them is correct and follow up personally to ensure they confirm. Please note that we cannot proceed your manuscript until we have received confirmations from all co-authors.) | One author (Lead study supervisor) is deceased (Late Md. Nazrul Islam Khan) (which was mentioned in the manuscript) and other two authors (also study supervisors) have retired recently and were on vacation! (Email’s IDs are checked and those are correct). | They are informed personally to response the e-mails and also informed about this review-response letter and sought some suggestions /helps where necessary. |
| 2. Funding information |  | “The authors received no specific funding for this work.” |
| 3. Competing interests |  | “Authors have no ‘Conflict of interests’ to declare” |
| 4. Full list of legends for all supporting information files (including figures, table and data files) after the references list |  | Mentioned after the reference list (one S1-table and S2:one excel appendix-figure-2 ) page 41 |
| 5. Suggestion of copyediting manuscript for language usage, spelling, and grammar thoroughly | At first, corresponding author revised and edited manuscript thoroughly according to the ‘review comments’ or the suggestions of the ‘Respective’ reviewers with necessary help/suggestions from co-authors. After that, in the presence of the corresponding author (as subject-matter of this manuscript is totally different for the copyeditor), the manuscript is thoroughly copyedited for language usage, spelling and grammar by Ms. Meherun Nessa, Associate Professor (English), Department of Language, Sher-e-Bangla Agricultural University, Dhaka-1207.  It takes several days for only copy- editing the manuscript by her. However, after computer editing, revision and rechecking by the corresponding author, this ‘Revised manuscript’ is prepared. | The manuscript is thoroughly copyedited (for language usage, spelling and grammar) by  Meherun Nessa, Associate Professor (English), Department of Language, Sher-e-Bangla Agricultural University, Dhaka-1207  E-mail: [meherun17@yahoo.com](mailto:meherun17@yahoo.com)  Acknowledgement of the copyediting service performed by Meherun Nessa, Associate Professor (English), is also mentioned in the ‘acknowledgement section’ of the manuscript. |
| Review comments to the authors (from two peer Reviewers) | **All addressed** | Corrected sentences/words or adding new words/sentences (always green)  Omitting the hyphenations (always yellow) |
| Reviewer 1  **Abstract**  * In the abstract, kindly guide the reader by putting subtopics e.g., introduction, methods, results, and conclusion. It is easy to follow when it has subtopics.  Indicate the number of female commercial sex workers who participated in your study and confidence interval of overall prevalence of STIS.  The language needs editing  in abstract (line 14), “About 41.8% (n=207/495) had any one infection (syphilis/or HBV)” | According to Journal criteria/guideline-- “Abstract should be conceptually divided into three sections (Background, Methodology/Principal Findings, and Conclusions/Significance), but not to apply these distinct headings to the abstract within the article file”.  In total, 495 FCSWs agreed to give 5 ml venous blood and overall prevalence rate of STIs was 43.6% (95% CI: 39.1-48.0). Majority (n=207/495) of them inflicted with either syphilis or hepatitis-B and a tiny portion (n=09/495) had co-infection. However, none of FCSW was positive for HIV.  Majority (n=207/495) of them inflicted with either syphilis or hepatitis-B | ‘Subheadings’ are inserted in the abstract and “Abstract with subheading” will be uploaded separately  Included in the abstract, in results (page 12)(including the table1, page 13) and in method section (page 6) (all highlighted)  The sentence in the abstract has changed/edited as and where necessary |
| **Introduction** |  |  |
| **LINE 42 and 45**  “During the period of 2020, 919 people were diagnosed with AIDS and Dhaka has the highest number of AIDS-patients (2,572) among high-risk-districts of the country.” Are these (2,572) only AIDS patients or both AIDS and HIV patients? Do they? I am asking because you follow your sentence with ….” Although, Bangladesh maintains <1% prevalence of HIV infection….” | These numbers (2,572) are only for the ‘AIDS patients’ (have AIDS defining illnesses to be referred to as AIDS patients), and During the period of 2020, 919 people were diagnosed newly with AIDS. Total HIV Positive patients (in 2020) was 14,000 (24% of them IDU)(From Reference number 8) | To inform the readers about the number of HIV infected patients we added a new sentence before this sentence of introduction on **page 2**  “Recently, AIDS related deaths have increased in Bangladesh and also posing the country at greater risks as 53% (7400) of HIV-positive cases remain missing, out of the total 14,000 detected cases [7,8]” |
| **Line 55 to 57**  “Ulcerative STIs (syphilis/cancroid/herpes simplex virus-2) increase 3-times whereas non-ulcerative STIs increases 2-times risk of HIV-transmission”. Kindly write its reference. | Actually, it has several references to cite together (as it mentioned 2 kinds of STDs i.e. ulcerative and non-ulcerative- under which several STIs remain). That’s why, the sentence is rearranged/paraphrased and 4 references (mentioned below) are included in the text and also in the ‘reference list’-to refer it fully. After adding 4 references (inserted at the ref. no.21, after Kakchapati et al (ref 20), the numbers of the whole reference list increased to 54. Number of the previous 21 increased to 24 (also all ref no. after 21 increased by 4). | New 4 References are written and highlighted (on the Page 3 and in the reference list, page 35)  Studies [21, 22, 23, 24] reported the presence of ulcerative STIs (syphilis/cancroid/herpes simplex virus-2) greatly increase the risk of acquiring or transmitting HIV infection than non-ulcerative STIs (gonorrhea/chlamydia) (by two to three times in some population).(page 3) |
| **New 4 references at reference list (page 35)** | 21. World Health Organization (WHO, 2016).Global Health Sector Strategy on Sexually Transmitted Infections 2016–2021. Towards Ending STIs (WHO-RHR-16.09-eng). https://apps.who.int/iris/rest/bitstreams/1032114/retrieve (Accessed September 2021)  22. Johnson LF, Lewis DA. The effect of genital tract infections on HIV-1 shedding in the genital tract: a systematic review and meta-analysis. *Sex Transm Dis*. 2008;35(11):946-959. doi:10.1097/OLQ.0b013e3181812d15  23. Looker KJ, Elmes JAR, Gottlieb SL, et al. Effect of HSV-2 infection on subsequent HIV acquisition: an updated systematic review and meta-analysis. *Lancet Infect Dis*. 2017;17(12):1303-1316. doi:10.1016/S1473-3099(17)30405-X  24. [Other STDs Increase Risk of HIV Infection - FHI 360.](https://www.fhi360.org/sites/default/files/webpages/Modules/STD/s1pg12.htm) *[Contraceptive Technology and reproductive health series.](https://www.fhi360.org/sites/default/files/webpages/Modules/STD/s1pg12.htm)* [Sexually transmitted diseases](https://www.fhi360.org/sites/default/files/webpages/Modules/STD/s1pg12.htm)*[.](https://www.fhi360.org/sites/default/files/webpages/Modules/STD/s1pg12.htm)*  <https://www.fhi360.org/sites/default/files/webpages/Modules/STD/s1pg12.htm> (Accessed September 2021) | |
| Line 64 up to 72  Too long sentence and it becomes unclear along the way. Split it into two sentences. |  | Done  (the long sentence divided in to 2 sentences, page 4) |
| Line 94, Line 125 and 376  etc.: Kindly, abbreviations do not start sentences. “FCSWs………….” |  | Done accordingly (page 5, 7, 21) page 23 (MLR), page 24 (HAI), ) |
| Line 117 : “ ………..and analysed in 2020 to find out how macro/micro structural-factors associated with the prevalence of STIs at physical, social, economic and policy levels”. Analyzed in 2020? Did you do the data analysis for this study in 2020? Or it is the primary data which was analysed in 2020? I think, it is the original research, which was analyzed in 2020, though I am not sure from the way you wrote it. Kindly rewrite it clearly | This study is original research, structural data (more than 50 variable) of which was analyzed in 2020. In fact, data (off course, primary data) of this study represents a part of a multi-level study about FCSWs (mentioned in the manuscript), other Data (Immuno-nutritional, infectious diseases and more) were analyzed biochemically/in laboratory and statistically after data collection. However, structural factors (especially sexual networking and HRSBs (except condom use), client related behaviours and some individual life-style risk behaviurs of FCSWS’s were analyzed in 2020 to find out the association of these variables with the prevalence of STI. | It is clearly Re-written in the method section(Page 6) |
| **Methods** |  |  |
| Line 146: Among the microphysical variables, for education, you have illiteracy and 1-12, what is 1-12? For people like me who do not know the education system of Bangladesh, 1-12 may not be clear to us. Is it lower (primary) level, middle (secondary) level, higher level of education (tertiary, university) level or all levels of education? | 1-12 means 1-12 years of schooling (as opposed to illiteracy rate). We mentioned together 1-12 years of education [(i.e. 1-5 years means primary, 6-8 years = junior, 9-10 years=secondary schooling certificate/SSC and 11-12 higher secondary certificate/HSC). Here very few had 6-8 years, 9-10 and 10-12 Y education and no one had university degree]. Overall, most (69.5%) were illiterate and few (30.5%) had academic education [(Mostly 26.9% (n=129) had primary (1-5 y) and together (6-12 y) only 4.4% (n=22) had Secondary education], so, for the sake of proper analysis we used only 2 categories (as data supported). | Mentioned (and highlighted) in method section (page 8) and also as ‘footnote’ under table 2 (Page 15). |
| **Analysis** |  |  |
| Overall, your statistical analysis and models are strong, and your results are compelling.  I recommend considering which models/results to highlight in this paper to make it easy for the reader. | Both adjusted models have similar important micro variables except for model-2 has extra 3 non-significant and 2 significant covariates (P<0.05). Statistically, model-2 has more power as overall-predicted-percentage/OPP and NR^2^/Nagelkerke R-square increased in model-2 from 76.8 to 79.2% and .492/49.2% to .530/53.0% respectively (as compared to model-1), may be due to remaining only one macro-policy factor (last one year forced sex) and one micro policy factor (condom from hotel boys) in the model-2 after controlling variables of model 1. This means, If ‘macro policy factor’ can be implemented it can alleviate other micro-level factors (individual, social, work environment and sexual networking) spontaneously. Overall, if all these variables (both macro and micro level factors) can be addressed in the interventions successfully, STIs can be minimized among FCSWs. However, model-1 represents existing situation i.e. micro-level factors predominant over macro-level. | It is mentioned in the result section (page, 18) and in the conclusion and recommendation (page 28-29 )(all highlighted) |
| Did you test for multicollinearity before building the regression model? It is not stated anywhere in your manuscript whether you did it or not, yet it is good to check the issue of multicollinearity every time before we build regression models. If yes, please mention it. | Yes, multicollinearity was observed before building the regression model though it is not mentioned.  Multicollinearity among independent variables was observed by using VIF/variation inflation factor and most of the covariates had VIF 1, and very few had <5. | In the method section (statistical analyses) Multicollinearity issue is mentioned and highlighted.  Page 10 |
| Line 185: I recommend you consider chronology of analysis e.g. We calculated the descriptive statistics to report on………… or We calculated the prevalence of STI among female commercial sexual workers……………. Then, we used bivariate analysis…………. From your results section (line 219), you started by reporting the prevalence of sexually transmitted Infections (STIs) among female commercial sex workers (FCSWs) in Dhaka city, Bangladesh. Then you followed it with bivariate analysis (line 257) however in your analysis section (line 182 ) bivariate analysis and prevalence of sexually transmitted Infections (STIs) do not appear anywhere. Kindly, try to be chronological and consistent. | **Edited sentences in Page 10 (2.6 statistical analysis)**  After screening and diagnostic conformation of the ‘prevalence of STIs’ (dependable variable/DV) among FCSWs, bivariate analysis i.e. cross-classification analysis (א^2^-test) and simple logistic regression (SLR) have been used to examine possible associations between STI-prevalence and different structural variables (or independent variables/IVs). P-value <0.05 was set as significance. ‘Multicollinearity test’ was observed (variation inflation factors/VIFs were mostly 1 or very few <5) among structural covariates before regression analyses. Multiple logistic regressions (MLR) were performed---- | Done and highlighted (the chronology of analysis) in the method section (statistical analyses).  Page 10 |
| Results:  Line 259: Would start this paragraph with this statement, bivariate analysis showed association between socio-economic, individual, and behavioral-risk-factors and STI-prevalence except for income-sharing. | **Edited sentence on the page 14**  Bivariate analyses showed significant (P<0.05) association between socio-economic, individual, and behavioral risk-factors with the STI-prevalence except for income sharing (table-2) | Done and highlighted (page 14). |
| Discussion Overall, I think the discussion is good. I propose you add interventions to reduce prevalence of STIS among female commercial sexual workers for each of the variables which were significantly associated with prevalence of STIS. What changes could be made to reduce the prevalence of STIS at the different levels in your discussion? |  | All are added in the conclusion and Recommendation section  (page 28-29) |
| **Reviewer#2: CORRECTIONS/EDITING/REVIEW** **Abstract**  Although, higher prevalence of STIs (216/495) was noticed among FCSWs, but none of FCSW was positive for HIV. Should be: Although, a higher prevalence of STIs (216/495) was noticed among FCSWs, but none of FCSW was positive for HIV. | **Result section of the abstract is rewritten:**  Overall, prevalence rate of STIs was 43.6% [.436(95% CI: 0.391-0.480)]. Majority (n=207/495) of them inflicted with either syphilis or hepatitis B and a tiny portion (n=09/495) had co-infection. However, none was positive for HIV | Some of the sentences in the abstract are corrected or rewritten/removed/rearranged in response to Reviewer 1 comment (highlighted) |
| **Line 1**... higher rates | **Line 1 (abstract)**  Female commercial sex workers **(**FCSWs) bear higher rates of sexually transmitted infections (STIs) | Corrected and highlighted with green |
| **Line 5** …The association of | **Line 5 and 6 (abstract)**  The association of structural determinants and STIs among FCSWs was not at the forefront of research earlier in Bangladesh | Corrected and highlighted with green |
| **Line 6** … at the forefront of |  | Corrected and highlighted with green |
| **Line 7 and 8----**individual risks and behavioural factors WITHOUT hyphen? | Without hyphens | All Corrected hyphenations are always highlighted with yellow |
| **Line 11----** macro/or micro… No / required | Abstract  Micro/micro structural factors  Or  Macro or micro structural factors | Done where necessary (abstract and all through manuscript and highlighted with yellow) |
| **Line 14--** ditto. No / required | Viral/bacterial | Corrected (abstract) |
| **Line 15 to 30--** need to check the syntax, grammar and too much use of hyphenation: macro-policy, micro-structural et cetera | Copyedited for syntax and grammar. | Removed all unnecessary hyphenations.  Copyedited By English language expert |
| Introduction and beyond: the excessive use of hyphenation continues transactional-sex key-populations/KPs permanent-clients Please review this. | Unnecessary use of hyphenation was removed (all through manuscript and highlighted with yellow colours ) | All these (i.e. transactional sex, KPs etc.) hyphenations (throughout the manuscript) are removed and highlighted with yellow colours. |
| **Line 47**  Men-sex-with-men (MSM)?? Should this be men-who-have –sex-with-men, with or without the hyphens?? | It should be Men who have sex with men (MSM) without hyphens | Corrected in the line 47 (page 3) and in other lines where the abbreviation MSM is used (page 31) (highlighted) |
| **Line 48-49**  Bangladesh may BE (??) on the brink of an | **Page 3**  Bangladesh may be on the brink of an HIV-epidemic | Corrected the sentence (page 3) |
| Some sentences were very difficult to understand, for example: Line 267 – 269  Interestingly, FCSWs used condom as 268 contraceptive had higher prevalence (49.5%) than who (32.0%) used pill/other modern269 methods (ligation/Nor-plant/copper-T/Injection) | Yes difficult! It was written according to table two, as percentages of two methods (pill%=21.8% and others%=10.2%) were added and written together (32%). Others means ligation/Nor-plant/copper-T/Injection) [those methods are mentioned under the table 2 as footnote’ by the sign ^¶¶¶¶^Others]  Interestingly, FCSWs used condom as contraceptive had higher prevalence rate (49.5%) than who used pill (21.8%) or other methods (10.2%) like ligation/Nor-plant/copper-T/Injection etc. (according to table2) | Sentence is corrected and highlighted (page 14) |
| Review conclusion: I was very interested in this paper after reading the abstract. However, it soon became clear that the paper needs major copy editing by the authors to improve the syntax, grammar, phrasing and, perhaps, to shorten the paper so that it is clear and succinct. I had no problems with the concept of the paper, methods and data gathered. I will leave decisive comments on the statistical analysis to those with better understanding of statistics than me.  I think the paper should be thoroughly revised and resubmitted |  | The manuscript is thoroughly revised according to the honorable Reviewer’s comments/suggestions and copyedited (for language usage, spelling and grammar) by  Meherun Nessa, Associate Professor (English), Department of Language, Sher-e-Bangla Agricultural University, Dhaka-1207  E-mail: meherun17@yahoo.com |
