# Supplementary material for: Social and structural determinants associated with the prevalence of sexually transmitted infections among female commercial sex workers in Dhaka City, Bangladesh
Source: PLOS Glob Public Health. 2024 Jan 18;4(1):e0002797. doi: 10.1371/journal.pgph.0002797 (PMC10796017; doi:10.1371/journal.pgph.0002797)
Supplement: Attachment — Submitted filename: Responses_To_Reviewers_Rebuttal_Letter.docx [file pgph.0002797.s005.docx]

**Table: Point to point responses to the Reviewers**

| **Comments** | **Explanations** | **Remarks** |
| --- | --- | --- |
| **Academic Editors** |  |  |
| Reviewers have agreed that this study is of public health importance, and the authors have improved the manuscript based on comments in the first round of the review. However, I agree with the reviewers that the manuscript is not written in standard English. The authors might need help from a more experienced researcher or English native speaker to condense the paper and correct typographical and grammatical errors throughout the text. | Thanks for the constructive comments. Sought help from a person (English speaker but not native) in public health (Public Policy Coordinator, Utah Academy of Nutrition and Dietetics. And RDN, LDN at Fresenius Medicare Care North America, USA) who read the article and showed some points (mostly identical to reviewers/editor’s comments and more). Following all suggestions  Strictly and spending quality time the article is edited thoroughly. | Tried to condense the paper and correct typographical and grammatical errors throughout the text according to Editor’s suggestions.  However, to shorten the article, ‘detailed categories of the Independent variables list’ (under Methods section) sets as supplementary file1. Texted on Page 6-7 |
| **6. Review Comments to the Author** |  |  |
| **Reviewer #1:** Much better than the first version. For readability and consistency, the article should be edited and updated by an English native speaker. There is too much use of abbreviations in this manuscript and the reader gets lost along the way.  “No HIV was found among FCSWs”, extract from the discussion. Can you explain or attach a reason why no HIV was found among FCSWs? | Yes, abbreviations were used. The abbreviation of ‘FCSWs’ (or ‘Female commercial sex workers’) was used 50-60 times in the manuscript; if not (4X60=260), 260 words will be increased, similar for STI/HIV/CLS/HRSBs, etc. | I tried to edit and update the article according to the honorable Reviewers’ and editor’s suggestions.  Tried to omit abbreviations throughout the manuscript (except FCSWs, STIs/HIV) and highlighted them with green. However, it increased word counts.  **No HIV:**  Explained with references in the discussion  “Donor support, government-led interventions, effective collaboration with implementing agencies, and peer education claimed to maintain a lower HIV prevalence rate in Bangladesh [1, 2].” Page 16 |
| **Reviewer #2:**  I think that this article has material of public health importance. However, I found the article a bit too long, wordy and still badly written. It needs stricter copy editing and shortening. Using simpler presented statistics and tables would do no harm either. |  | Shortened, copyedited strictly, as necessary.  Tried to explain the results with simple statistics in the Discussion. However, the  Tables are shortened in the usual format.  To shorten the article, Some information (under the Methods section) sets as supplementary file1 |
