# Supplementary material for: Social and structural determinants associated with the prevalence of sexually transmitted infections among female commercial sex workers in Dhaka City, Bangladesh
Source: PLOS Glob Public Health. 2024 Jan 18;4(1):e0002797. doi: 10.1371/journal.pgph.0002797 (PMC10796017; doi:10.1371/journal.pgph.0002797)
Supplement: Attachment — Submitted filename: Response to Reviewers_Rebuttal_letter.docx [file pgph.0002797.s006.docx]

**Table: “Point to point” response letter to the reviewers**

| **Review Comments** | **Explanation/Clarifications** | **Remarks** |
| --- | --- | --- |
| **Reviewer #1** |  |  |
| Reviewer #1: Thank you. for addressing all the comments raised in the last review. The English is much better than the previous one. Though there are some statements which are not clear to the reader. You can do one more edit again. |  | Thanks. I have addressed all of your valuable comments. |
| **Title**- Kindly edit the title by removing capital letters in the middle of the sentence. |  | All capital letters are removed from the title. |
| “High-risk behaviors' such as monthly coitus with permanent clients (AOR/.33), group sex ever (AOR/2.1), heterosexual anal intercourse ever (AOR/.03), and consistent condom use (AOR/.13) were also significant predictors of STIs”. Why do you say that “consistent condom use” is a high risky sexual behavior? | “Consistent condoms use”/CCU is not a high-risk sexual behavior; instead, it is a protective measure against STI-  **First:** In the multiple logistic regression model (Table 4) adjusted odds ratio/AOR of consistent condom use was 0.13 [here OR <1, protective factor, an odds ratio of less than 1 implies the odds of the event happening (STI prevalence) in the exposed group (Condom use group) are less than in the non-exposed (non-use group) group] which refers to CCU is **87% less likely to be** associated with STIs as compared to Inconsistent/No use of condoms.  **Secondly:** here reference category (R) is Inconsistent/No use of condoms or non-exposed group (shown in table 4).  However, in group sex, the odds ratio was 2.1 (here, OR is >1, which implies **greater odds of the event happening in the exposed versus the non-exposed group**), meaning group sex is two times more likely to be associated with STIs. | The sentence is written clearly, addressing the "odds ratio" as less than one (<1).  High-risk behaviors such as monthly coitus with regular clients (AOR/.33), no anal sex ever (AOR/.03), and consistent condom use (AOR/.13) were less likely to be associated with STIs. In contrast, the association of group sex ever (P<0.05) with STIs was reported to double (AOR/2.1). |
| 'Work environment' like sex on roads/parks/shrines/markets (AOR/2.6) and HIV-testing ever (AOR/2.5) also correlated with STIs (P<0.05). “HIV-testing ever” does not fit in the 'Work environment' group. A work environment is the setting in which you perform your job, therefore “HIV-testing ever” seems misplaced. | “A work environment is the setting in which one performs a job”- of course, authors agree with this statement. However, we are talking about FCSW’s ‘work environment’ where ‘HIV-risk’ (HIV-testing as well) is a fundamental criterion to be considered for keeping them free from STIs.  Shannon and colleagues (2014) offered a ‘structural HIV-determinants framework,' where ‘HIV-testing’ is an essential characteristic under the ‘work environment’ domain (Shannon et al., 2014; figure 1, page 176; Reference no. 40,) |  |
| …….and micro-policy factor, ‘getting condoms from hotel boys’ (AOR/.34). ‘Getting condoms from hotel boys’ is not clear. | It refers to access to condoms; hotel boys were one of the condom sources among other sources (e.g., from colleagues, self-buying, NGOs) mainly for hotel-based FCSWs (table 3, 4) | Written clearly |
| Socio-economic/Individual risk factors: Behind the sex work. What does “Behind the sex work” mean? | It means the reasons for the ‘sex work’ or reasons for being ‘sex workers’ | Corrected and written clearly |
| In data analysis, did you do multivariate analysis or multivariable analysis after bivariate analysis? From your description, you did multivariable logistic regression, not multivariate. Kindly check and clarify this. | Thanks for this comment. Indeed, we did “multivariable analysis” (which was multiple logistic regression, also mentioned in the statistical analysis section) as there exists only one dependable variable/DV (i.e., STIs) (although authors mistakenly wrote ‘multivariate analysis’ in the result section, which means >2 DVs and multiple independent variables) | Corrected and highlighted. |
| **Reviewer #3:** |  |  |
| Reviewer 3: This is the 3rd version of a manuscript that aims to identify social and structural factors associated with prevalent STIs among female sex workers in Bangladesh. The authors have made a valiant attempt to respond to earlier critiques; however, additional revisions are needed. As I was not one of the former reviewers, I unfortunately have **some new concerns** **regarding presentation and interpretation of results**, as well as some concerns with language as indicated below. |  | I have addressed all of your valuable comments. Thanks. |
| **Language**. The authors took to heart comments from the reviewers and had their paper revised by a native English speaker. However, there are still numerous grammatical issues. |  | Tried to solve some of the grammatical issues. |
| The authors have reduced the number of abbreviations, but several still remain that are not commonly used (e.g., HRSB, GS, HAIs, CCU, SLRs, PCs). Please do not create your own abbreviations. |  | Abbreviations like HRSB, GS, HAIs, CCU, SLRs, and PCs are removed. |
| In several places, the language used is pejorative and stigmatizing (e.g., ‘male homeless street beggars’, page 18 and ‘lower tier FCSWs,' page 18 ). The authors are urged to use person-centered language that is no-stigmatizing. |  | Corrected on page 18 |
| Throughout the paper, language is used that infers causality (e.g., ‘predictors’), but this is a cross-sectional analysis so causal inferences cannot be drawn. The authors should refer to their associations as ‘**correlates**’. On page 14, change the term ‘correlated with’ to ‘associated with’. |  | All associations are written, showing the variables were associated with STI (as ‘**correlates**’).  On page 14, the term ‘correlated with’ is changed, written as ‘more likely to be associated with’. |
| In several places, the authors use the term ‘prevalence rate’. Prevalence is a proportion, not a rate. Please remove the word ‘rate’ from these sentences. |  | ‘Rate’ was removed from the sentences and written correctly.  Page 10, page 16 |
| The authors use the phrase ‘permanent clients’ but permanence seems too strong. I suggest ‘regular clients’ instead. |  | “Permanent clients” are replaced with ‘regular clients.' |
| The last sentence in the abstract does not make sense as written. |  | The sentence is removed from the abstract. |
| Top of Page 6. Data were likely ‘reconstructed’ not ‘re-arranged’. |  | Included in the method section (pages 5-6) |
| “Dalal” need to be more clearly defined. |  | Defined in the text (page 12), and also as table’s footnote (page 13) |
| Bottom of page 9. The sentence beginning with “When family members…” does not make sense. |  | The sentence is corrected:  Sex workers were more infected with STIs if family members knew about their professional “sex work”.  (Page 10) |
| Table 2. The heading ‘Behind sex work” does not make sense. |  | Removed and written correctly (Reasons for being sex workers). (Page 11) |
| Methodology. The authors categorize several of their variables as macro or micro at various levels. However, in some cases, these levels are inappropriate. For example, being forced to have sex in the last year is referred to as ‘macro-policy’ which does not make sense.  Similarly, how is entering the sex trade through ‘poverty/deception/others’ classified as ‘macro-physical’? Poverty is a macro-economic factor whereas deception would be micro-social.  **In table 2, the phrase ‘micro-physical’** is beside “individual risks/behavioral’. Which is it? The authors are encouraged to use categories already assigned by Shannon et al (Lancet 2015), or Strathdee et al (Lancet 2010). | This paper followed some well-known works of literature (Urada et al., 2013; Pitpitan et al.,2013; Shannon et al., 2014 and 2015) on various structural factors of HIV/STI risk environment.  (Also mentioned in the conceptual framework, page 8).    Urada et al. (2013, reference no. 37, introduction chapter) clearly defined that venue type,  and histories of physical/sexual/emotional abuse are micro-physical factors.  Urada et al. (2013, reference no. 37) clearly defined force/  Deception into sex work as a macro-physical factor (introduction).  As 55.8% of FCSWs entered into this profession because of poverty (Macro-economic), followed by 26.1% for deception (macro-physical); thus, 'factors for being the sex workers’ denoted as Macro-Physical factor  ‘**micro-physical’** was (mistakenly) misplaced. | Forced sex/policing/violence  are included as a micro-social factor    ‘micro-physical’ is placed beneath its previous position. Page 11 |
| It is not clear how the authors could infer that qualitative data on syphilis could be used to indicate active infection, which relies on quantitative titers. | In this study, both non-treponemal (VDRL/venereal disease research laboratory test) and treponemal (**TPHA/** Treponema pallidum hemagglutination) tests were performed (by one of the authors) (already described in the section 2.5 laboratory method, page 7) for the screening (VDRL) and conformation (TPHA) of syphilis. Moreover, a rapid test (Immuno-chromatographic strip) was used for robust conformation.  Here VDRL/RPR are qualitative tests, and **TPHA**, a diagnostic test, is the gold standard for the diagnosis of syphilis as compared to RPR/VDRL test. TPHA is used to detect the dissolved amount of antibodies in a patient's serum sample against the causative agents of syphilis.  The rapid plasma reagin/RPR tests (same as a non-treponemal test) can be used instead of VDRL. Authors used VDRL. | As described in section 2.5 laboratory method, page 7 |
| On page 9, under the heading "Individual Risk Factors," the authors include several social-structural variables that are not intrinsic to the individual (e.g., internal migration). |  | The heading is written correctly (4.2.1 social and individual risk factors), and the sub-headings (social and individual) are separated. (pages 9-10) |
| Exact p-values should be reported in the Tables. |  | Exact p-values were included in all tables except for the table 4 (where all P<0.001 are P=.000). However, actual p-values of all significant nine variables (P<0.05) of adjusted model-1 are mentioned in the abstract and discussion (page 17-19) |
| Table 4, the final multivariate model, includes several variables (e.g., group sex) that are not statistically significant. Why? | “group sex”  Remained significant in all 3 models (unadjusted model-1, adjusted model-1, and hierarchical-adjusted model-2) | Nine variables [Age (p=.031), years in the sex trade (p=.000), condoms as contraceptives (p=.046), monthly coitus with regular clients (p=.036), group sex ever (p=.030), Anal sex ever (p=.000), consistent condom use (p=.000), trading sex on-street/parks/elsewhere (p=.030), and HIV-testing ever (p=.000)] were significant in the final adjusted model-1. |
| Discussion. The Discussion should not focus on variables that were not statistically significant in the final model. |  | In the discussion,  those variables (nine) statistically significant in the final adjusted model-1 were discussed. |
| This sentence does not make sense: “The successful implementation of ‘macro-level factors’ can create pathways to alleviate micro-level factors.” |  | The sentence was removed from the conclusion and recommendation. |
| Limitations should include mention that 140 FCSWs were excluded since they refused to provide lab samples, which is a selection bias. |  | One hundred and forty refusal cases were mentioned in the limitation. |

.
