# Supplementary material for: Social and structural determinants associated with the prevalence of sexually transmitted infections among female commercial sex workers in Dhaka City, Bangladesh
Source: PLOS Glob Public Health. 2024 Jan 18;4(1):e0002797. doi: 10.1371/journal.pgph.0002797 (PMC10796017; doi:10.1371/journal.pgph.0002797)
Supplement: Attachment — Submitted filename: Response_To_Reviewer_Rebuttal _letter.docx [file pgph.0002797.s007.docx]

**Table: point to point responses to the reviewer’s comments**

| **Reviewer’s comments** | **Explanation/clarifications** | **Remarks** |
| --- | --- | --- |
| Reviewer #4:  Overall comments  This study purports to be the first to examine correlations between STIs and structural determinants in Bangladesh. The authors report some interesting findings. However, there are various elements of the methods, discussion, and other parts of the manuscript that I believe require editing and/or clarification before the paper can be seriously considered for publication. I apologize to the authors, as I know they have already revised and resubmitted this manuscript based on feedback from a prior reviewer, and I know it is frustrating to have new concerns raised by a new reviewer. My detailed comments follow. |  | Thanks for the meticulous review and all these valuable suggestions.   - All Done |
| General  • Based on the literature I'm familiar with, it seems unusual for a paper focused on "sexually transmitted infections” to only include Hepatitis B and syphilis. I would suggest that the authors make it clear in the title and/or early on in the manuscript that the focus here is narrowly on these two infections. The authors should also provide a justification for why these 2 are the focus. | - This study screened two viral STIs, HIV and Hepatitis B, among FCSWs, which are scanty in Bangladesh. And also screened for bacterial spirochete infections like syphilis |  |
| • I suggest that the authors use an equal sign rather than a backslash when reporting odds ratios (i.e., AOR=x rather than AOR/x). |  | - Thanks. All odds ratios (OR) are reported by the equal signs (=) throughout the manuscript. |
| Abstract  • Suggest moving the 3rd sentence about lack of prior research in Bangladesh up to be the second sentence. |  | - Thanks. Moved the sentences as suggested. |
| • In the sentence that begins, “In total, 495 FCSWs…” the authors should say “screened for Hepatitis B and Syphilis” rather than STIs broadly. |  | - Done   (screened for HIV, hepatitis B, and syphilis) |
| • In the sentence that begins “Most FCSWs were inflicted…” the word “inflicted” implies they were deliberately infected, so I suggest another word. |  | - The word has been changed.   (inflicted replaced with infected) |
| • When reporting the multivariable logistic regression results, for age and years in the sex industry, the authors should mention the reference group. | - In this study, eleven (n=11) categorical variables were found to be significantly (P<0.05) associated with STIs in an adjusted model (multivariable analysis). However, in the abstract, there is a word limit (250 words). Thus, it is impossible to mention the "reference group" for all variables while narrating them (which is also narrated in the result section for Table 4). | - Reference groups were mentioned in the text (results page 10, 13,14) |
| • In the sentence about high-risk behaviors, including “no anal sex” and “consistent condom use," is confusing, as these are the opposite of high-risk. I suggest either reorienting these variables throughout the manuscript/analysis to be "anal sex" and "inconsistent condom use" or not calling these "high risk behaviors." | - By observing the odds ratio (whether it is >1=more likely or <1=less likely), the sentences described in the abstract seem all right (the way odds ratio <1 was described in the literature). While described in the text, no anal sex means its reference category is 'yes'. Tables 3 and 4 are well described. - No anal sex ever (AOR=.03, P<0.001, (^R^) Yes), and consistent condom use (AOR=.13, P<0.001, (^R^) Inconsistent/never) were less likely to be associated with STIs.(page 15) - By Inverting the odds ratio (1/AOR), we can say that Anal sex is 7.69 more likely to be associated with STI or no anal sex is 97% less likely (described in discussion page 20) | - Sentences are also re-written in the results (page 15) |
| • It is unclear what “monthly coitus with regular clients” means and compared to way.  Is this at least monthly on average, within the past month, monthly vs. less frequent, monthly vs. more frequent? Please clarify. | - There were 3-types of relationship with regular clients: (1) Monthly-coitus (2) fortnightly-coitus (3) and Weekly-coitus. However, only monthly coitus was significant in the bivariate analysis and ultimately remained in the multi-level analysis (Table 3, 4) - How many times (weekly/fortnightly/monthly) in a month and how frequently (1-2 times/ >3 coitus per session) did FCSWs meet with the regular clients |  |
| • Instead of “last year’s forced-sex,” I suggest saying “experiencing forced sex in the past year” and removing the quotation marks. |  | Written “experiencing forced sex in the past year” (with removing quotation mark) |
| • What does “condom source” mean and what is the reference group to which the AOR corresponds? | “condom source” means places/persons from where FCSWs managed to collect/get a condom, the reference group was Colleagues | - Used ‘condom collected from’   (also in table 4, page 16) |
| • In the last sentence, why are there quotation marks around “commercial sex work?” |  | Removed inverted commas from commercial sex work |
| - Introduction - • I would suggest the authors consider restructuring the introduction to start with basic epidemiology of HIV/STIs in FCSW in Bangladesh, then introduce the structural determinants framework and then use that framework to present what is already known about drivers of STIs, etc. |  | - Restructured |
| - In the sentence that begins, “Recently, new AIDS cases…” when the authors say 53% of cases remain “missing,” do they mean “undetected?” It’s not clear what “missing” means. | - Missing means nobody (including the Government registry) knows their whereabouts after being detected for AIDS. Because of social stigma/bad remarks or even hatred, most AIDS patients (many of them are migrant workers and have wives or legal partners) remain absconding. | - Sentence re-written.   (---as 53% (7400 out of 14,000) of HIV-positive detected cases remain missing.) |
| - The sentence that starts, “Bangladesh may be on the brink…” is confusing, and it is unclear how the second part of the sentence demonstrates that the country is on the brink of an HIV epidemic. |  | - Re-written |
| - In the last sentence of the first paragraph, please expand and explain how the criminalization of sex work can accelerate the spread of STIs/HIV into the general population. | - Due to having multi-partners concurrency (legal partners, emotional/non-paying partners, paying partners, etc., and more importantly, having drug addict clients/partners) of sex workers, STIs have already been transmitted into the general population (references: WHO, Global, HIV, Hepatitis, and STIs program.   (https://www.who.int/teams/global-hiv-hepatitis-and-stis-programmes/populations/sex-workers#:~:text=Modelling%20studies%20indicate%20that%20decriminalizing,reduction%20in%20new%20HIV%20infections.) |  |
| - Remove quotation marks around “criminalization of sex work.” |  | - Removed the quotation marks. (page 4, 1^st^ para) |
| - In the last paragraph, the authors mention the “illegal nature of sex work.” It would be helpful to say whether sex work is fully criminalized, whether some aspects are legal, etc. | It's tough to say whether sex work is fully criminalized or whether some aspects are legal- it varies from country to country, population to population. Society and laws are different in different countries. However, **Globally-**  Sex workers face high stigma and criminalization almost everywhere (WHO). Modeling studies indicate that decriminalizing sex work could lead to a 46% reduction in new HIV infections in sex workers over ten years while eliminating sexual violence against sex workers could lead to a 20% reduction in new HIV infections (WHO). (https://www.who.int/teams/global-hiv-hepatitis-and-stis-programmes/populations/sex-workers#:~:text=Modelling%20studies%20indicate%20that%20decriminalizing,reduction%20in%20new%20HIV%20infections.  **(**Sex Workers Network (SWN), Bangladesh and Sex Workers and Allies in South Asia (SWASA) (Ref 4 in manuscript)  (Global Network of sex work project (nswp, [www.nswp.org](http://www.nswp.org))   - Authors mentioned in reference (14, 26) |  |
| - There is no need for quotation marks around "STI prevalence."   and I would suggest changing this terminology as prevalence may have population-level implications when the manuscript is examining associations with STIs within individuals. | - This cross-sectional study screened/investigated two viral STIs (HIV, Hepatitis B) and one bacterial STI (Syphilis) among female sex workers/FCSWs. Unfortunately, HIV was not detected among FCSWs; thus, STI prevalence (43.6%, 95% CI 39.1-48.0) for the remaining 2 STIs was used. - Cross-sectional studies, also known as prevalence studies, examine the data on disease and exposure at one particular time point. - This study used a standard formula (Reference 42) to calculate the sample size, which was the 'Representative sample' of the population (e.g. FCSWs). - Inference was made about the population from the sample, using Confidence Interval (CI) to quantify the uncertainty. Moreover, the Z-statistic (for the confidence level, the z value is 1.96) indicates the number of standard deviations an observation is above or below the 'population' mean. For the conventional 95 % confidence level, the z value is 1.96 since 95 % of a normal distribution would lie within ±1.96 standard deviations on either side of the mean. | - Quotation mark removed from STI prevalence. |
| Materials and Methods   - I am confused about the study population. Were they participants of a previous study who were recontacted to participate in a new study? - Or is there just one study with the present manuscript drawing on different data from that study than a previous manuscript did?   Please clarify. | - Not at all. - One study with the present manuscript restructured some lifestyle data. A previous manuscript was published on Nutritional status/BMI (Reference no. 42) from different/BMI data of the same study. - Authors previously mentioned (page 6, 1^st^ para) that it was a multi-level study (DETAILED ARE: data collected on family members, children's health and Nutrition, FCSW’s health (BMI), morbidity, lifestyles including details of sexual life (risky sex, group sex, anal sex), FCSW’s clients, intimate partners, faced lifetime problems (police arrest, forced sex, snatching/sharing money, etc.). Blood specimens also covered their infections/STIs, Nutrition (serum protein, albumin), Immunity (IgG, IgA, IgM), and immuno-nutrition (serum vitamin A, E and serum copper, zinc, iron). - However, in 2020, authors took an endeavor to analyze lifestyle data in the structural format (independent variables) to see how these structural data influence their infectious status. |  |
| - While Supplementary File 1 offers some additional information about the measures used, there are some things that remain unclear even there. I also think many readers are unlikely to refer to that document so some additional details within the main manuscript are needed. | - Supplementary File 1 contains the information that was previously incorporated in the 'main manuscript'. However, the authors were asked (previous review) to cut short the manuscript as it was wordy. - Thus, that information was provided as supplementary. |  |
| - Outcome variable: Based on my reading of the manuscript, the outcome variable would be better described as a positive test for Hepatitis B or Syphilis. |  | - Edited the outcome variable as a positive test for Hepatitis B or Syphilis (page 7) |
| - Independent variables that are not clear based on current wording/level of detail: “behind the sex work,” “seeking customers to another city," and "income sharing.” Please briefly explain what these mean in the text. | - “Behind the sex work” means the reasons for being sex workers (macro-level factor only significant in bivariate analysis). - Seeking customers in another city (macro-social factor ) means whether FCSWs go to another city to catch customers (social mobility) - Shared income with others (either willingly or forcefully), already described under Table 2 as footnotes (^§^Local muscle men/family/police/others) | - Corrected “Behind the sex work” in the text and written as “Reasons for being sex workers” (page 12, table 2) - Corrected “income sharing” in the text and written as “shared income with others."   ( willingly or forcefully) page 12 |
| - For sexual networking and high-risk sexual behaviors, some variables do not include a period. Does "non-paying partner" mean currently, ever, within a particular period of time? What is the time period for coitus with regular clients and oral sex? | - In the questionnaire, there were a series of questions regarding partners (2 types: 1. paying 2. Non-paying) and clients (2 types: 1. casual/temporary 2. regular/permanent/steady)   All paying and Non-paying partners denotes a current relationship   - Last week’s oral sex (mentioned in the table 3, page 14) | All paying and Non-paying partners had a current relationship.  Corrected in the text (page 7 and table 3, page 14) |
| - I would move up the description of consistent condom use to the area where the condom use measure is first mentioned. |  | Description of the consistent condom use is moved on to page 7 from page 8 under the heading (2) sexual networking and high-risk--- |
| - What does “lifetime problems” mean? | Any problem [police arrest, rape or forced sex, abuse- (verbal, physical), snatching money etc.] faced by any FCSW in her lifetime while working as a sex worker. The previous manuscript also described these under the table footnotes. |  |
| - Is forced sex by a particular perpetrator? In the Supplementary file, it says “forced sex/violence,” so please clarify what is included here. | - In the questionnaire, forced sex, violence, abuse, etc. were mentioned separately, and only forced sex was significant (P<0.05) in multi-level analysis. - Violence was written mistakenly and was not significant in bivariate analysis. - In this study, forced sex was performed by police, local goons/hoodlums, and even sometimes by the clients (mentioned under the table's footnote) | The word Violence is removed. |
| - Estimation of consistent condom use: Suggest rewording this to something like “Participants were classified as engaging in consistent condom use if they reported that condoms were used during their most recent encounters of vaginal, anal, and oral sex with all commercial and non-commercial sexual partners.” |  | Done (Rewording for the estimation of consistent condom use, page 8 ) |
| - Laboratory methods: I’m not sure that this level of detail is necessary, but if it was requested by a prior reviewer, I defer to them. | Both screening (Qualitative) and confirmation (Quantitative) of HIV, Hepatitis B, and syphilis (all methods were sophisticated, expensive, laborious, and needed special protection because these organisms, especially HIV and hepatitis B were highly infectious/ contagious) were performed. Thus, short laboratory methods were provided within a paragraph for better understanding. |  |
| - What does “qualitative determination” mean? | In a laboratory for microbiological analysis, the 'qualitative technique’ provides ‘yes’ (presence of bacteria/virus) or ‘no’ answers. Contrarily, quantitative determination (also confirmatory) provides numerical information (i.e., number and percentage of reads) using various machines. |  |
| - In the statistical analysis section, the sentence about VIFs, is this reporting results of a multicollinearity test or describing methods? | A variance inflation factor/VIF is a tool for identifying the degree of multicollinearity among multiple independent variables in a multiple regression model. Moreover, mentioning VIF or tolerance (1/VIF) is a formal, at the same time, a better way to express multicollinearity. However, one can just mention that a multicollinearity test is performed and no multicollinearity is observed during analysis. |  |
| - Please explain hierarchical logistic regression, as not all readers may be familiar with this. | Hierarchical logistic regression is a framework for model comparison of nested regression models (rather than a statistical method). It can be useful for evaluating the contributions of previously entered predictors or independent variables (IVs), as a means of statistical control, and for examining incremental validity if variables of interest explain a statistically significant amount of variance in the dependent variable (DV) after accounting for all other IVs. | The description of hierarchical logistic regression is incorporated in the 2.6 statistical analysis section (page 9) |
| • I suggest describing the conceptual framework much earlier, before the description of measures, so that the measures can be clearly situated within the framework. |  | Done.  Conceptual framework (page 6) is placed before the description of measure (page 7) |
| • *All of the references used in the “*conceptual framework” section seem to be from other settings.  Is there any literature from Bangladesh that led to this hypothesized framework? If so, please add.  If not, I suggest the authors state that the framework is based on work from other settings that is hypothesized to be applicable to Bangladesh as well. | - The authors followed the structural framework of Shannon et al. (2014, ref 40) to rearrange the structural variable of this study. - As mentioned earlier in the manuscript (page 5), no study in Bangladesh worked on structural factors before. - Already mentioned in the manuscript (Conceptual Frame Work, page 6; and **2.3 Measure**, page 7) |  |
| Results  • In the paragraph just before Table 1, sentence that begins “Sex workers were more infected…” I do not think that the authors mean they were more infected and suggest changing to “more likely to be infected.” |  | Once again, Thanks, done (page 10) |
| • In Table 1, I do not understand what the p-values correspond to, as there does not appear to be any comparison or test, just a presentation of the prevalence of Hep B and syphilis. |  | P-values (the column) are removed from the Table 1 (page 11) |
| • Table 2: P-values cannot equal 0, though they may be infinitesimally small. Please edit to be p<0.001. | Previously (earlier in all manuscripts except for the last), p-values (in Table 2, 3) were written in the standard format (if P=0.000, it was denoted as P<0.001, if P=0.04 it was P<0.05),; at the last review authors were asked to put the actual p-values instead.  These have been changed/edited further. | All P-trends in table 2 and 3 are re-written (on pages 12, and 14) |
| • Table 2 and Table 3: For binary variables, I suggest rather than having two lines, one with the variable and one with "yes" or "no" (e.g., migrated in from other cities, did not seek customers in another city), only the first line is included in the table. | - As authors have to deal with numerous variables, to keep/adjust the same type of variables on the same page, some variable categories were written in short spaces/on 2 lines. However, it is corrected/re-written. | - Corrected (table 2, page 12) |
| • For the education variable, is everyone necessarily illiterate if they never attended school and is everyone who ever attended school literate? It is confusing to me why the categories are illiterate vs. any schooling rather than no schooling vs. any schooling. | - Yes, those who attended school are likely literate, and those who could not afford it (schooling) are illiterate, except for some of them who could sign/write their names. | No schooling vs. schooling years (Education category) has been added (page 12, table 2) |
| • In the sexual networking section second sentence, I think the word “partners” should be added after “non-paying.” |  | Thanks. The word ‘partners’ is added after non-paying. |
| • Here again I have a question about the meaning of “monthly coitus,” but if it clarified earlier as suggested this may be fine. | - This study observed that Regular clients met their clients at different time points. There were 3 types of relationship with regular clients (1) Monthly-coitus (2) Fortnightly-coitus (3) and Weekly-coitus with regular clients. However, only monthly coitus was significant in bivariate analysis (model1), and ultimately remained in the multi-level analysis models (models 2 and 3). |  |
| • In the work environments section, why are quotation marks used around certain words and phrases? |  | Quotation removed from some words of the work environments section (page 8) |
| • Table 3: Are the options under “access to condoms or condoms collected from” mutually exclusive? Couldn’t the respondent have gotten condoms from more than one source, or is the question specific to the last time they accessed condoms? | - Yes, both are mutually exclusive (Both have four categories), but in this study, ‘Condoms collected from’ is more straightforward/clear than the other one. 'Access to condom' is used by Shannon et al. (ref 40) for their structural model. - This question (which was asked generally to FCSWs, not specific to the last time), was set as an open-ended in the questionnaire, and after getting all answers, they were codded and then entered into the computer for analysis. Thus, in this study, FCSWs did not get condoms from multiple sources. - Note that if some questions had dual answers, these were analyzed by using the 'multiple response' option in SPSS. |  |
| • Multivariable analysis: add “Table 4” in the first set of parentheses. |  | - Added and rearranged (page 15) |
| • Should include reference group for categorical variables like age and years in sex work. |  | - Included reference category(^R^) to all categorical variables (page 15) |
| • Various comments like those related to “high risk behaviors” and presentation of AOR apply to this section as well. |  | - Yes, Applied as suggested (page 15) |
| Discussion  • Overall, I think the authors could do a more thorough job of interpreting their results in the context of prior literature and the intervention implications. | - The authors have done a vigorous literature (>100 manuscripts) review to find out relevant literature for results (eleven significant variables). However, not all variables are found as such, as there exists variation in methods, population, and period (already mentioned in the manuscript), though some phenomena/variables like risky sexual behaviors (like vaginal sex, anal sex, condom use etc.) have interrelated aspects. However, in the previous manuscripts, authors detailed but asked to cut short. |  |
| • In the first paragraph, sentence beginning “However, participants of this study…” add the word “which” after “data collection.” |  | The word "which" is added after data collection (page 17) |
| • Why have donor agency interventions been hampered? | Lack of Proper Government Policy or strategy/liaison with the donor agencies could be responsible, For example:  1. Closure of the Family Health International 360 (fhi360) HIV prevention program since July 2014. (Ref 2)  2. Funding declined significantly from the project of Global Fund and Health Population, Nutrition, Service Delivery Program (HPNSDP) for 'Harm Reduction services" (Reference 2 and its cross reference 21.( Govt. of Bangladesh, Revised National Strategic Plan for HIV and AIDS Response, 2011-2017. 2014.)  Or, maybe for the transitional period time gap to renew the previous one. | Sentence re-written (page 17) |
| • First line on page 17, it’s not clear what the authors are referring to when they write “contribute in both ways.” | “Contribute in both ways” means contribute for both clients and FCSWs as they exist in parallel around the structural factor that can promote or reduce factors related to FCSWs and clients. Actually, the authors followed the framework (Figure 1, page 176 on the journal page, Shannon et al., Curr Opin HIV AIDS 2014, 9:174–182 DOI:10.1097/COH.0000000000000042) offered by Shannon and colleagues (2014) (reference 40), which is a complex model of multiple interacting variables, but easy for describing if visualized. The framework depicts how structural HIV determinants in sex work are produced through a dynamic interplay of macro-structural factors (e.g., social, economic, and health policies, and laws governing sex work, mobility and migration of sex workers and clients, geography and sociopolitical transitions, stigma, cultural norms on gender and  sexuality); the community organization of sex work (e.g., community empowerment, sex work collectivization); and the physical, social, economic, and policy features of work environments (e.g., venue-based characteristics, managerial practices, local  policing, coverage and access of condoms, HIV/STI testing, HAART)   - According to that figure, structural HIV determinants can promote or reduce interpersonal factors (e.g., partner-level/dyad-level risks and protections, such as condom negotiation, sexual networks, and patterning) and interact with individual factors of sex workers, their clients, and their intimate, non-paying partners, including behavioral (e.g., drug use, duration in sex work), biological (e.g., age, sex, race, HIV characteristics, STI co-infection)(Shannon et al, Curr Opin HIV AIDS 2014). | Sentence re-written (page 18) |
| • In the next sentence, please expand/explain on what is meant by and what evidence there is for ONLY macro policy supporting laws leading to successful interventional outcomes. | - A systematic review (ref 45) revealed that if intervention is not supported by macro-policy factors (laws), it diminishes promising health-promoting interventions. | References in the texts clearly stated that macro policy-supporting laws leading to successful interventional outcomes (page 18) |
| • In the discussion about contraceptive type, a number of questions arose for me. Could respondents choose more than one option? If not, how would a person answer who uses both respond? Also, I think the authors should dig a little deeper into the literature to help explain this finding of condom use as contraceptives being associated with STIs. | - Like the 'condom source', this (contraceptive type) question was also an open-ended type in the questionnaire, and after getting all answers they were codded and then entered into the computer for analysis. Thus, in this study, FCSWs used only one method of contraception.   Very few articles show findings of condom use as contraceptives being associated with STIs.   - Already mentioned in the manuscript that most FCSWs treated 'condoms' as a means of 'contraception', not for preventing STIs/HIV [ref: 12,47,50]. - Thus, setting this in mind, whenever they used condoms as a contraceptive, most FCSWs did it incorrectly/inconsistently. However, some pill users may have the opposite mindset about condoms. Thus, they used condoms correctly and constantly to get dual protection. | - Another finding in an earlier study in 2001 added (Ref 35)(page 18) |
| The bivariate association is not significant, but once it is put into a model that also includes other variables, including consistent condom use, a significant association emerges. How can we interpret this and what are the implications? | **NOTE:** In bivariate analysis, most of the variables were significant (Already mentioned in the statistical analysis (page 8), and in the text (page 9: result section), and also shown in table 2 and table 3 (Table 2 and 3 for Bivariate and table 3 for multi-level analysis). Standard procedures for logistic regression were performed. It is notable that most of the variables were significant in Bivariate analysis except for Income sharing (table 2, page 11), oral sex, having membership of the sex worker's organization, and being controlled by a pimp (all in Table 3, page 13). After that all significant variables (P<0.05) were put together in the model to perform multivariable analysis. |  |
| • I do not understand the last sentence of this paragraph related to unmet reproductive health needs. | Female commercial sex workers (ref 6, 12, 44, 46, 50), especially Asian FCSWs (6, 12, 44) did not meet their sexual and reproductive health needs (Potential unmet need for modern contraceptives i.e. pill, intrauterine device, and family planning services) and experienced unwanted pregnancies, abortion complications, pre, and post-pregnancies complications etc.  , | Detailed in the text (Page 19) |
| • Page 18, 3rd line: Do you mean less or more condomless anal sex? Please clarify. | - Writing two sentences together created confusion; it is now written as   However, higher monthly coital-frequencies with intimate partners were reported to be associated with less condom usage (P<0.05) [47], and increased risk of unprotected heterosexual anal intercourse (HAI) [31]. | Re-written (page 19) |
| • In the sentence that begins “An earlier study described that…” the word “indulged” has some potentially negative connotations. Suggest using “engaged” as a more neutral word. |  | - Neutral word ‘engaged’ is used   (page 19) |
| • The meaning of the last sentence on page 18 is unclear. Are the authors taking this from their findings  or prior literature and what is the purpose here? | Yes, which was included in detail in the previous manuscript but removed. Authors experienced (in collecting and analyzing data) that ---  Interviewing and analyzing the data of this study regarding CCU, the authors noticed that the 'perception of CCU' was very poorly understood by the FCSWs (69.4% of them were illiterate). If they used a condom every six clients out of seven, they thought it to be enough for protecting STI/HIV, did not even count one 'unprotected event', while one unprotected event happened with the intimate  partner/steady client may be resulted in transmission of STIs from self to the partners or  one partner to other partners or to the bridging population, thereby creating a complex convoluted  ‘web of transmission’. Recent studies also reported that condomless sex/inconsistent condom use is a higher risk factor for a new STI case among Bangladeshi FCSWs [6,26].   - Sharing study finding which is not found in prior literature. | (Page 20) |
| • Work environment section page 19: The last sentence implies that FCSWs face violence because they are illiterate and destitute, which I don’t think is supported by the literature. Please rephrase and/or explain. | - All over the world, sex workers, especially CSWs from Asia/Lower middle-income countries, faced violence (References 4,5,12,29). Being illiterate and destitute may be two reasons for experiencing violence among many other causes, especially for Asian FCSWS (ref 3, 4, Sex Workers Network (SWN), Bangladesh and Sex Workers and Allies in South Asia (SWASA) | - Rephrased (page 21) |
| - I’d like to see more discussion about the findings around prior HIV testing being associated with STIs. What could explain this?   Are FCSWs at high-risk of HIV/STIs more likely to get tested for HIV/other STIs but not Hep B and Syphilis so their Hep B/Syphilis might have gone undetected so this is really a reflection of risk rather than a true association between testing and infection?  Or are they getting tested and diagnosed but having trouble accessing treatment? Or some other explanation? What are the implications? | - This study showed that prior HIV testing among FCSWs was associated with STIs, however very few research is related to this finding, this may be due to- - FCSWs are at higher risk for being infected with STI/HIV. However, being tested Positive especially for HIV is related to stigma and social exclusion, (mentioned in the manuscript), so most of the FCSWs (both groups) did not perform HIV-testing, but remarkable portion of them would be positive if they get tested. When some of them tested for HIV, they are concerned about their infection status/STIs which is consistent with Hossain et al (ref 54) and showed--   Having previous history of STI-testing were 6.6 times more likely to have HIV-testing [ref 54].   - As standard procedure was maintained in data collection (randomization, multistage sampling) and analysis (categorization, logistic regression), so prior HIV-Testing is definitely associated with STI status but not the cause (cross-sectional nature). However, direct cause is unknown, may need longitudinal study. - Usually, if tested for HIV positive, FCSWs do not get trouble accessing treatment as HIV treatment is free since (in 80s when 1^st^ HIV was detected in Bangladesh) the inception of ‘HIV prevention program’. | - One finding (ref 54) is included (page 21) |
| - First line of page 20, what “both groups” are the authors referring to? | 1) Hotel-based FCSWs, and 2) On-street or floating sex workers (worked on road/park/Shrine/shop/elsewhere) | Name of the two groups are added within bracket (page 22) |
| • I’d like to see some implications/recommendations more closely/directly tied to the study’s results. |  | Additional recommendations closely tied to the study results are incorporated (page 22) |
| • Additional limitations: only testing for Hep B and syphilis and not other STIs like chlamydia and gonorrhea certainly limits the ability |  | Additional Limitations are added (page 22) |
| Figure 2  • The numbers at the bottom of the chart are not readable and need to be adjusted. |  | Figure 2  At the bottom of figure2, Numerical ranges (of the chart) were changed (0--0.5, 1.0-- 1.5, and so on) |
| - Please add a vertical line at the odds ratio of 1 so readers can easily see which ORs are above and below that. |  | - Done (A vertical red line is drawn on the position of the odds ratio/OR 1) |
